# The abnormal expression of circ-ARAP2 promotes ESCC progression through regulating miR-761/FOXM1 axis-mediated stemness and the endothelial–mesenchymal transition

**DOI:** 10.1186/s12967-022-03507-3

**Published:** 2022-07-16

**Authors:** Pei Xu, Lei Wang, Qingtao Liu, Pengkai Gao, Fengqing Hu, Xiao Xie, Lianyong Jiang, Rui Bi, Fangbao Ding, Qi Yang, Haibo Xiao

**Affiliations:** Department of Cardiothoracic Surgery, Xinhua Hospital Affiliated to Shanghai Jiao Tong University School of Medicine, No 1665 Kongjiang Road, Yangpu District, Shanghai, 200092 China

**Keywords:** Circ-ARAP2, Esophageal squamous cell carcinomas, miR-761, FOXM1, Cancer stem cell

## Abstract

Circular RNAs (circRNAs) belong to a novel class of noncoding RNA that gained more attention in human cancer pathogenesis. The role of circRNA in esophageal squamous cell carcinoma (ESCC) is largely unclear. Present investigation was to characterize new circRNAs regulating ESCC progression and explore the regulatory mechanisms in ESCC. In this study, circRNAs differentially expressed in ESCC and adjacent normal tissues were characterized via high-throughput sequencing. Then the differentially expressed circRNA between ESCC and adjacent normal tissues were investigated using Rt-qPCR. The role of circ-ARAP2 expression on tumor progression were detected in both in vivo and in vitro. Luciferase reporter assays were used to identify the relationships among circ-ARAP2, microRNA (miR)-761 and the cell cycle regulator Forkhead Box M1 (FOXM1). The result of the expression profile analyses regarding human circRNAs in ESCC demonstrated that circ-ARAP2 was up-regulated significantly in both ESCC tissues and cell lines. Downregulation circ-ARAP2 suppressed ESCC proliferation, tumor growth and metastasis in both in vivo and in vitro. The data also suggested that miR-761 and FOXM1 were circ-ARAP2 downstream targets which were confirmed through luciferase reporter analysis. Overexpression of FOXM1 or inhibiting miR-761 restored ESCC cell proliferation and invasion ability after silencing circ-ARAP2. The study also found that circ-ARAP2 influenced the endothelial–mesenchymal transition (EMT) and cancer stem cells differently by regulating miR-761/FOXM1. In one word, the results demonstrated that abnormal circ-ARAP2 expression promoted ESCC progression by regulating miR-761/FOXM1 axis-mediated stemness and EMT.

**Supplementary Information:**

The online version contains supplementary material available at 10.1186/s12967-022-03507-3.

## Introduction

Esophageal carcinoma is a severe malignancy with poor prognosis and mortality [1]. ESCC is a frequent type of esophageal cancer, occurring in 90% cases. Because of recurrence, extensive metastasis and invasion, the overall ESCC 5-year survival rate is < 13% after initial diagnosis [2, 3]. Esophageal cancer is divided into five stages according to TNM stage, namely stage 0, stage I, stage II, stage III and stage IV. In the past, there were simply three clinical stages: early stage is equivalent to stage 0 to stage I, which refers to patients with tumor invasion of mucosa and submucosa, tumor less than 3 cm, and no local lymph node metastasis; Metaphase is equivalent to stage II to III, until the tumor has invaded the muscularis and adventitia of patients; Advanced stage IV refers to patients with distant lymph node metastasis and distant metastasis [4]. Although surgery is therapeutically useful in the early stages of ESCC, most patients are diagnosed in the late stages of the disease, when common therapeutic methods, including surgery, chemotherapy and radiotherapy, are not effective enough to inhibit recurrence [5]. Accumulation studies confirmed that ESCC is one of the most common malignant tumors worldwide and is characterized by promotes cancer stem cell (CSC) features and induces epithelial-mesenchymal transition (EMT) [6–8]. The study also found that EMT plays a critical role in the development of drug resistance in multiple cancer types. Besides, it equips cancer cells with CSC-like characters that also are associated with chemotherapy resistance [9]. Therefore, early diagnosis is essential to advance treatments and reduce mortality regarding ESCC patients. It is urgent that we discover new ESCC therapy targets as well as efficient diagnostic markers.

circRNAs are in a new family of ncRNA that function importantly in human malignant tumors [10, 11]. Nevertheless, biology functions of circRNAs remain largely unknown, particularly in cancer pathogenesis. Investigations suggested that many circRNAs contain microRNA (miRNA) binding sites, which might regulate miRNA downstream targets through sponging and arresting miRNA activities [12, 13].

circRNAs belong to a class of recently discovered RNAs which are expressed extensively in eukaryotic cells. The advancement of high-throughput sequencing and technologies significantly improved our understanding of circRNAs [14, 15]. Recently, we discovered that the circRNA ARFGAP with RhoGAP domain, ankyrin repeat and PH domain 2 (circ-ARAP2) was expressed abnormally in ESCC patients, but its role and regulatory mechanism in ESCC progression was unclear. The present study verified biology function of circ-ARAP2 along with its underlying molecular mechanisms in ESCC development from several aspects, which may help identify new biomarkers for ESCC.

## Materials and methods

### Ethics statement

We acquired four fresh ESCC tissues with paired adjacent non-cancerous tissues (within an area of 3 cm around the tumors) after first obtaining informed consent from patients in Xinhua Hospital of Shanghai Jiaotong University, China. Patients received no radiotherapy or chemotherapy prior to tissue sampling according to previous studies [16]. Our team snap-froze samples in liquid nitrogen and maintained them at − 80 °C before RNA extraction. The Ethics Committee at Xinhua Hospital of Shanghai Jiaotong University oversaw the investigation.

### Animals

Our team leveraged BALB/c nude mice (n = 12) with four weeks old weighing 15–20 g (SLARC, Shanghai, China). The Ethics Committee at Xinhua Hospital affiliated with Shanghai Jiaotong University approved the study.

### Cell culture and treatment

Our lab purchased esophageal cancer cell lines ECA-109R, TE-1, TE-1R, KYSE-150, ECA-109 and KYSE-150R from the Cell Bank at the Chinese Academy of Sciences. The cells were cultured in RPMI-1640 (Gibco, Grand Island, NY, USA) supplied with fetal bovine serum (Lot: 217396RP; FBS; Gibco) of 10%, 100 U/mL penicillin and 100 mg/mL streptomycin (Lot: 15,140–122; Gibco) at 37 °C in an incubator containing 95% air and 5% CO_2_.

For in vitro experiments, we transfected siRNA against circ-ARAP2 (si-circ-ARAP2), miR-761 inhibitors, a Forkhead Box M1 (FOXM1) overexpression vector or negative controls (GeneChem, Shanghai, China) into cultured KYSE-150R or ECA-109R cells. For in vivo experiments, a lentiviral-stabilized circ-ARAP2 silencing vector (sh-circ-ARAP2; GeneChem, Shanghai, China) was stably transfected into KYSE-150R cells.

### Bioinformatics analyses

Our team determined circRNA/miRNA target genes through *CircularRNA Interactome*. Our team predicted interactions between mRNA and miRNA through *TargetScanHuman*.

### Clonal formation assays and cell proliferation

Cell proliferation was assayed utilizing Cell Counting Kit-8 (Lot: C0038; CCK-8; Invitrogen, Carlsbad, CA, USA). Transfected cells were seeded into 96-well plates in triplicate targeting 2000 cells/well, and viability was assayed at 0, 1, 2, 3, and 4 days after seeding, following the manufacturer instructions. We also assayed colony formation by transfected cells seeded into plates having six wells at 2000 cells/well and grown in DMEM with FBS of 10% for 10 days. Colonies were calculated and photographed after fixation and staining.

### Flow cytometry cell cycle assays

Cells were fixed in 70% ethanol overnight at 4 °C, then resuspended in staining solution (Lot: P00096; Beyotime, Shanghai, China) for 0.5 h at 4 °C. Statistician analyzed cells that stained by flow cytometry (Beckman Coulter).

### Fluorescence in situ hybridization (FISH)

Our team employed circ-ARAP2-specific Cy3-labeled probes for FISH Zhang, et al. [17]. Technician counterstained nuclei using 4,6-diamidino-2-phenylindole (DAPI). Our lab conducted experiments through kits from Genepharma (Shanghai, China).

### Quantitative reverse transcription-polymerase chain reaction (qRT-qPCR)

Technician gained RNA via TRIzol reagent (Lot: 15,596–026; Invitrogen) and cDNA was synthesized via pTRUEscript First Strand cDNA Synthesis Kit (Lot: 170–8890; Aidlab, Beijing, China). Our team did qRT-qPCR with 2 × SYBR Green qPCR Mix (Lot: PC3301; Invitrogen) using ABI 7900HT qPCR system (Thermo Fisher Scientific, Waltham, MA, USA) and measured expression fold-change following the 2^−ΔΔCT^ method. Technician performed qRT-PCR amplification with following primers: circ-ARAP2 forward: 5′-GTACCAGAGATTCCAGGGTC-3′, reverse: 5′-CTTCACAGTACTGCTTTAC-3′; miR-761 forward: 5′-ACAGCAGGCACAGAC-3′, reverse: 5′-GAGCAGGCTGGAGAA-3′; FOXM1 forward: 5′-ACGTCCCCAAGCCAGGCTC-3′, reverse: 5′-CTATGTAGCTCAGGAATAA-3′; U6 forward: 5′-CTCGCTTCGGCAGCACA-3′, reverse: 5′-AACGCTTCATTTGCGT-3′; GAPDH forward: 5′-AATCCCATCACCATCTTCC-3′, reverse: 5′-CATCACGCCACAGTTTCC-3′. We normalized FOXM1 and circ-ARAP2 expression levels to GAPDH, and normalized the miR-761 expression level to U6.

### Tumor sphere formation assays

KYSE-150R and ECA-109R cells were harvested and resuspended as single cells in serum-free medium. Following cell counting, technician seeded 200 cells/well in 200 μL serum-free medium in plates with 96 wells, 10 wells/group, altering the medium every 2 days. Our lab photographed 5 regions randomly selected from every group of wells with camera-equipped microplate reader (Leica, Wetzlar, Germany) and computed the sphere percent as the count of spheres/200. The spheres with diameters > 50 μm (cutoff size) were counted and the diameters of sphere were analyzed.

### Transwell assays

For Transwell migration assays, our laboratory resuspended 2 × 10^4^ cells in 200 μL serum-free culture medium and added them to upper chambers of a Transwell plate. For invasion assays, technician precoated the Transwell chambers with Matrigel (Lot: 3,356,234; BD Biosciences, San Jose, CA, USA) and put equal numbers of cells to upper chambers. Our team then put 500 μL DMEM containing 15% FBS to the lower chambers. Technician erased the cells in the upper chamber after incubation for 1 d and cells that had migrated to or invaded the lower membrane surface were fixed with paraformaldehyde of 4% and stained with Crystal Violet solution of 0.1%. Our lab then photographed and counted the cells that invaded or migrated.

### Luciferase reporter assays

We utilized pGL3-reporter luciferase vector (Lot: E1751; Promega, Madison, WI, USA) to construct pGL3-FOXM1 3′-UTR-WT, pGL3-FOXM1 3′-UTR-Mut, pGL3-circ-ARAP2-WT and pGL3-circ-ARAP2-Mut vectors. Technician seeded 293T cells in 24-well culture plates and cultured them for one day before co-transfection with WT or Mut FOXM1 3′-UTR/circ-ARAP2 and miR-761 mimics or negative control (miR-NC) following the manufacturer recommendations. Our team monitored luciferase activity 2 d after transfection by Dual-Luciferase Reporter Assay System (Promega) and calculated the firefly luciferase ratio for every transfected well.

### Tumor xenograft formation and metastasis assays

Tumor xenograft formation was examined after injection of 2 × 10^7^ viable KYSE-150R cells stably expressing sh-NC or sh-circ-ARAP2 into right flank of nude mice. Technician calculated tumor sizes every 5 d utilizing vernier calipers and computed tumor volume by 1/2 × length × width^2^. After 1 month, technician euthanatized mice for further experiments.

For metastasis analyses, technician transfected KYSE-150R cells (2 × 10^5^) with sh-NC or sh-circ-ARAP2 luciferase expression vectors and injected them intravenously into mice tails. After 1 month, our lab analyzed KYSE-150R cell metastasis using bioluminescence imaging following intravenous luciferin injection (150 mg luciferin/kg body weight) into the mice tails.

### Immunohistochemistry

Tissue samples were fixed in 4% paraformaldehyde, embedded them in paraffin and sectioned. Our team then incubated the tissue sections with anti-Ki-67 primary antibody at 4 °C overnight, followed by horseradish peroxidase-conjugated secondary antibody.

### Statistical analyses

Results are represented as means ± standard deviation (SD). GraphPad Prism (GraphPad, La Jolla, CA, USA) was leveraged to compute differences between groups. *P*-values ≤ 0.05 were regarded as statistical significance.

## Results

### High circ-ARAP2 expression plays a role in ESCC

**Fig. 1:**
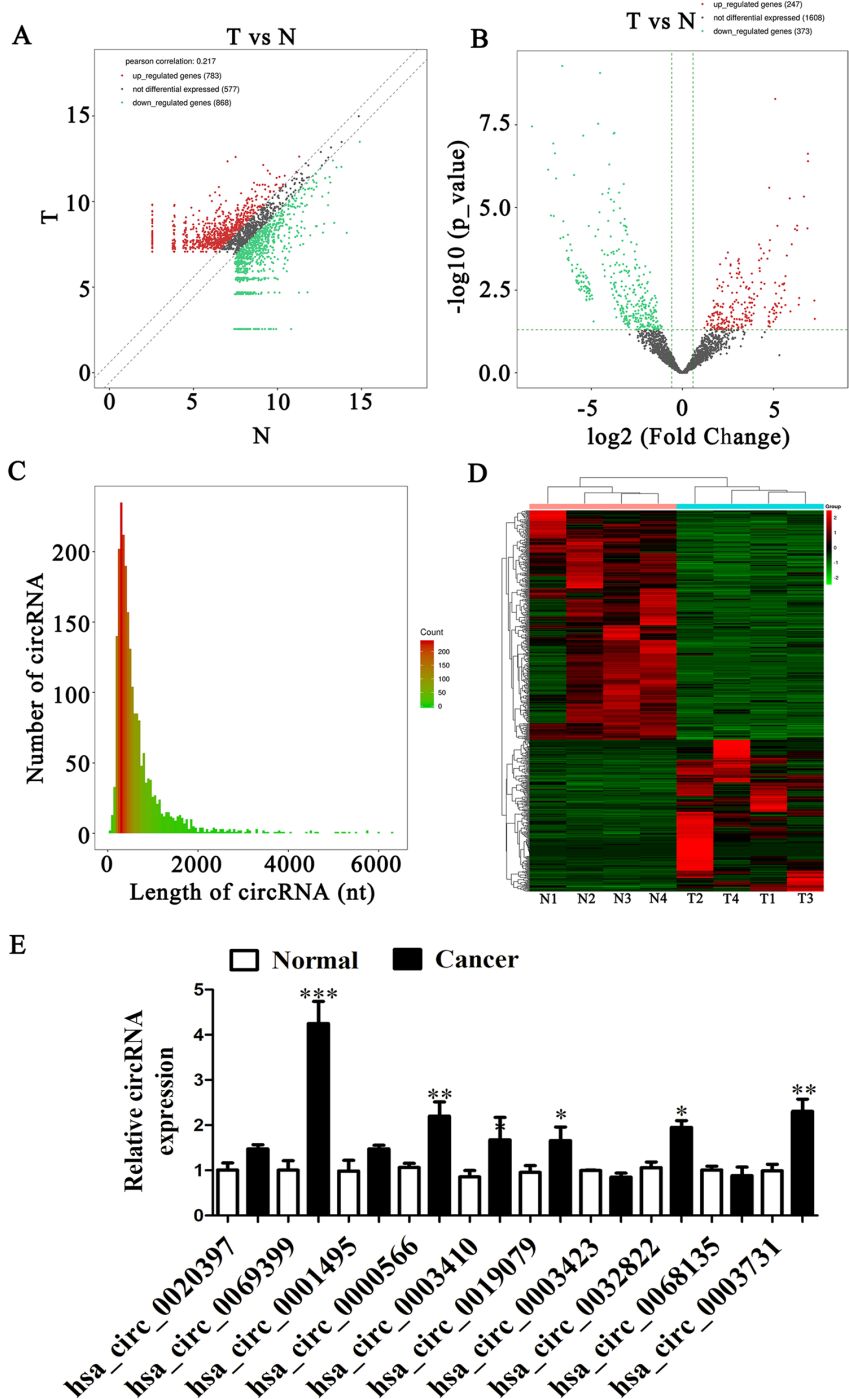
The differential expression of circRNA in esophageal squamous cell carcinomas (ESCC). **A** Scatter plots used to evaluate the differential expression of circRNAs between ESCC tissues and the matched non-tumor tissues. **B** Volcano plots used to visualize the differential expression between two different groups. x-axis: log_2_ ratio of the level of circRNA expression between normal and tumor tissues. y-axis: the false discovery rate value (-log_10_ transformed) of the circRNAs. **C** Length distribution of the identified circRNAs. x-axis: the length of the circRNAs detected in this study. y-axis: the abundance of circRNAs classified by different lengths. **D** Heat map of all the differentially expressed circRNAs between normal and tumor tissues. **E** Relative expression of the 10 indicated circRNAs from ESCC tumor tissues and adjacent non-tumor tissues listed as measured by RT-qPCR. The data are presented as the mean ± SD. *p < 0.05; **p < 0.01. N, non-tumor tissues; T, tumor tissues

High-throughput sequencing of four ESCC tissues and non-cancerous adjacent tissues found that 783 genes were up-regulated and 865 genes were down-regulated (Fig. 1A) in cancerous tissues. Volcano plot analysis determined that 247 circRNAs were also up-regulated and 373 circRNAs were down-regulated (Fig. 1B) in same tissues. The study also found that the identified circRNA length distribution was approximately in the range of 300–500 nt (Fig. 1C). Heat maps show all of differentially expressed circRNAs in tumor and normal tissues (Fig. 1D). Differentially expressed circRNAs are given in Additional file 1. RT-qPCR was employed to validate the ten most highly up-regulated circRNA expressions: hsa_circ_0020397, hsa_circ_0069399, hsa_circ_0001495, hsa_circ_0000566, hsa_circ_0003410, hsa_circ_0019079, hsa_circ_0003423, hsa_circ_0032822, hsa_circ_0068135 and hsa_circ_0003731. Data illustrated that hsa_circ_0069399 expression incremented significantly in tumor tissues comparing to matched non-cancerous tissues (Fig. 1E).

**Fig. 2:**
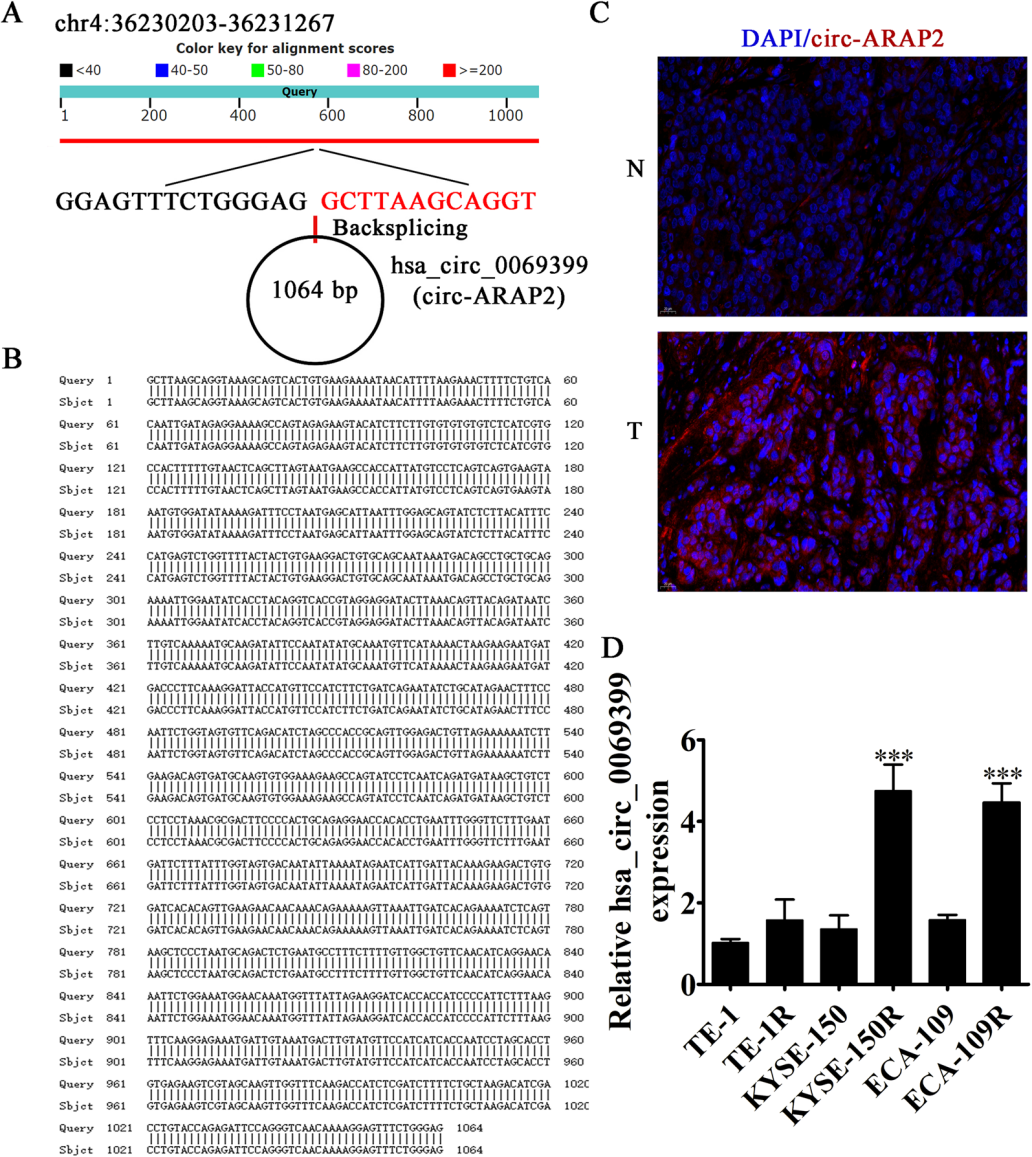
The genomic loci and subcellular localization of circ-ARAP2. **A** The genomic loci of the ARAP2 gene and circ-ARAP2. **B** The sequence of circ-ARAP2. **C** Fluorescence in situ hybridization (FISH) detection show the subcellular localization of circ-ARAP2 in KYSE-150R. **D** RT-qPCR detection show the expression of circ-ARAP2 in TE-1, TE-1R, KYSE-150, KYSE-150R, ECA-109, ECA-109R. Data are presented as the mean ± SD. ^***^p < 0.001 vs. TE-1

The hsa_circ_0069399 originates from the *ARAP2* gene exon with 1064 bp and is located in chr4:36,230,203–36,231,267. Therefore, we renamed hsa_circ_0069399 as circ-ARAP2 (Fig. 2A, B). FISH experiments showed that circ-ARAP2 expression was incremented in ESCC tissues comparing with matched non-tumorous tissues, and the data also suggested that circ-ARAP2 was localized primarily to cytoplasm (Fig. 2C). RT-qPCR results demonstrated that circ-ARAP2 was expressed in ESCC cell lines TE-1R, TE-1, ECA-109R, KYSE-150R, KYSE-150 and ECA-109. The data also showed that ECA-109R and ECA-109R had the highest circ-ARAP2 expression (Fig. 2D). Consequently, we selected KYSE-150R and ECA-109R cells for further study.

### Down-regulation of circ-ARAP2 suppressed ESCC proliferation and tumor growth in vivo and in vitro

**Fig. 3:**
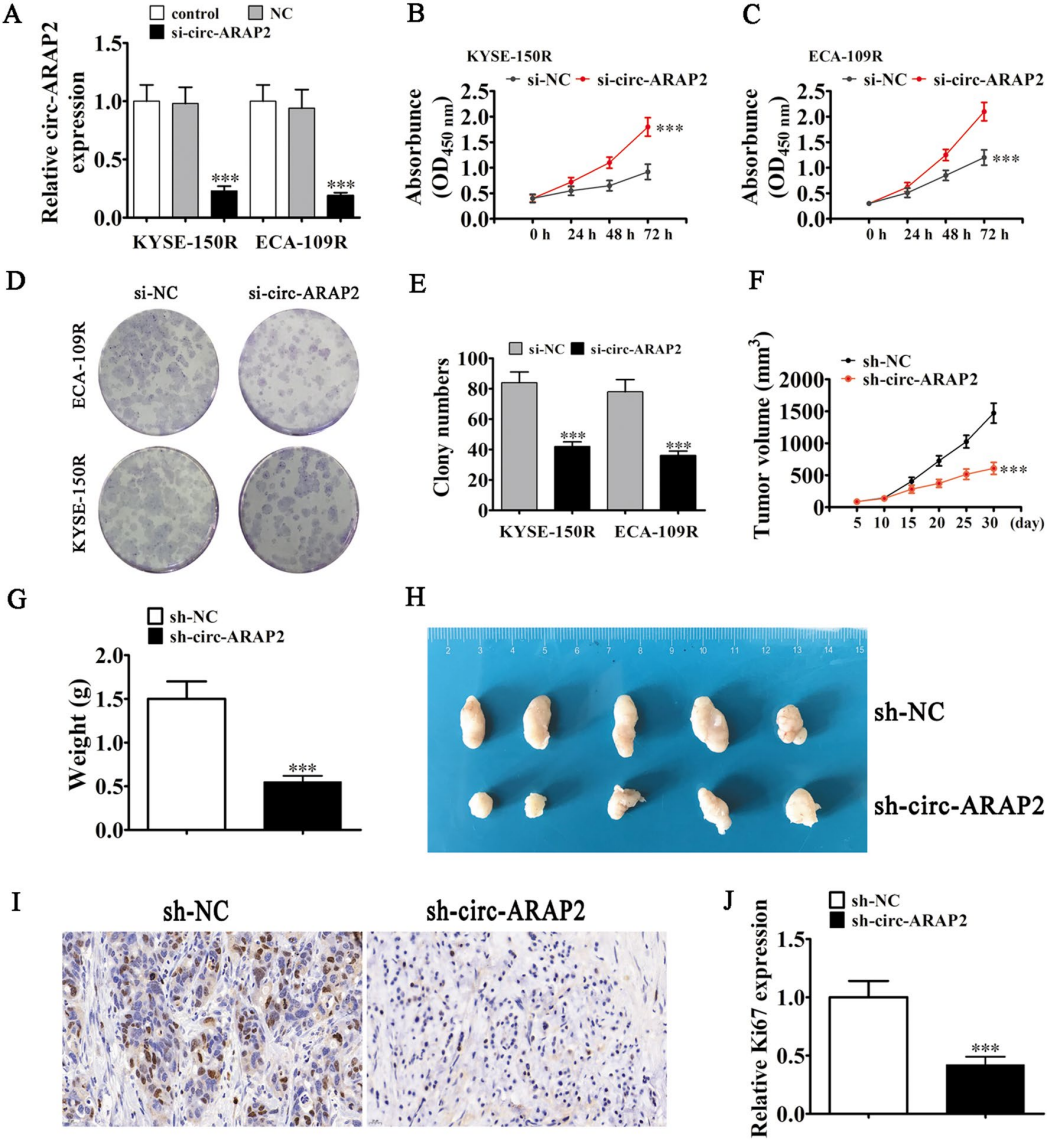
Downregulation circ-ARAP2 suppressed ESCC proliferation and tumor growth in both in vitro and in vivo. **A** RT-qPCR detection show the expression of circ-ARAP2 after transfected with siRNA against circ-ARAP2 (si-circ-ARAP2) or negative control (si-NC). Data are presented as the mean ± SD. ^***^p < 0.001 vs. control. **B**, **C** CCK8 detection show the effect of circ-ARAP2 to proliferation of KYSE-150R and ECA-109R. Data are presented as the mean ± SD. ^***^p < 0.001 vs. si-NC. **D**, **E** Cloning formation assay showing the cellular proliferation in both KYSE-150R and ECA-109R cells. Data are presented as the mean ± SD. ^***^p < 0.001 vs. si-NC. **F**–**H** Representative photographs of KYSE-150R tumor formation in the xenografts of nude mice.Summary of the tumor volume and in mice that were measured weekly. Data are presented as the mean ± SD. **p < 0.01; ***p < 0.001 vs. si-nc. Tumor weight was measured 30 days post-injection. Data are presented as the mean ± sd. ***p < 0.001 vs. sh-NC. **I**, **J** Immunohistochemical analysis shows the percentage of Ki-67-positive cells. Data are presented as the mean ± SD. ***P < 0.001 vs. sh-NC

In order to clarify the role of circ-ARAP2 during ESCC progression, siRNA against circ-ARAP2 were constructed and transfected it into both ECA-109R and ECA-109R cells. RT-qPCR results found that circ-ARAP2 expression was significantly down-regulated after circ-ARAP2 was silenced in both ECA-109R and ECA-109R cells (Fig. 3A). CCK8 detection (Fig. 3B, C) and clonal formation assays (Fig. 3D, E) showed that cellular proliferation was significantly decreased in both KYSE-150R and ECA-109R cells.

Then a xenograft mouse model were constructed to detect circ-ARAP2 effects on tumor growth in vivo. KYSE-150R cells expressing a lentiviral-stabilized circ-ARAP2 silencing vector (sh-circ-ARAP2) or a negative control (sh-NC) were used in this study. One month after transplantation, we harvested the xenografts. Results showed that circ-ARAP2 silencing suppressed tumor growth in volume and weight (Fig. 3F–H). Immunohistochemical detection showed that Ki67 expression was decreased in the circ-ARAP2-silenced group (Fig. 3H–J). These results suggested that down-regulation of circ-ARAP2 suppressed ESCC proliferation along with tumor growth in vivo and in vitro.

### The circ-ARAP2 knockdown suppressed ESCC metastasis

**Fig. 4:**
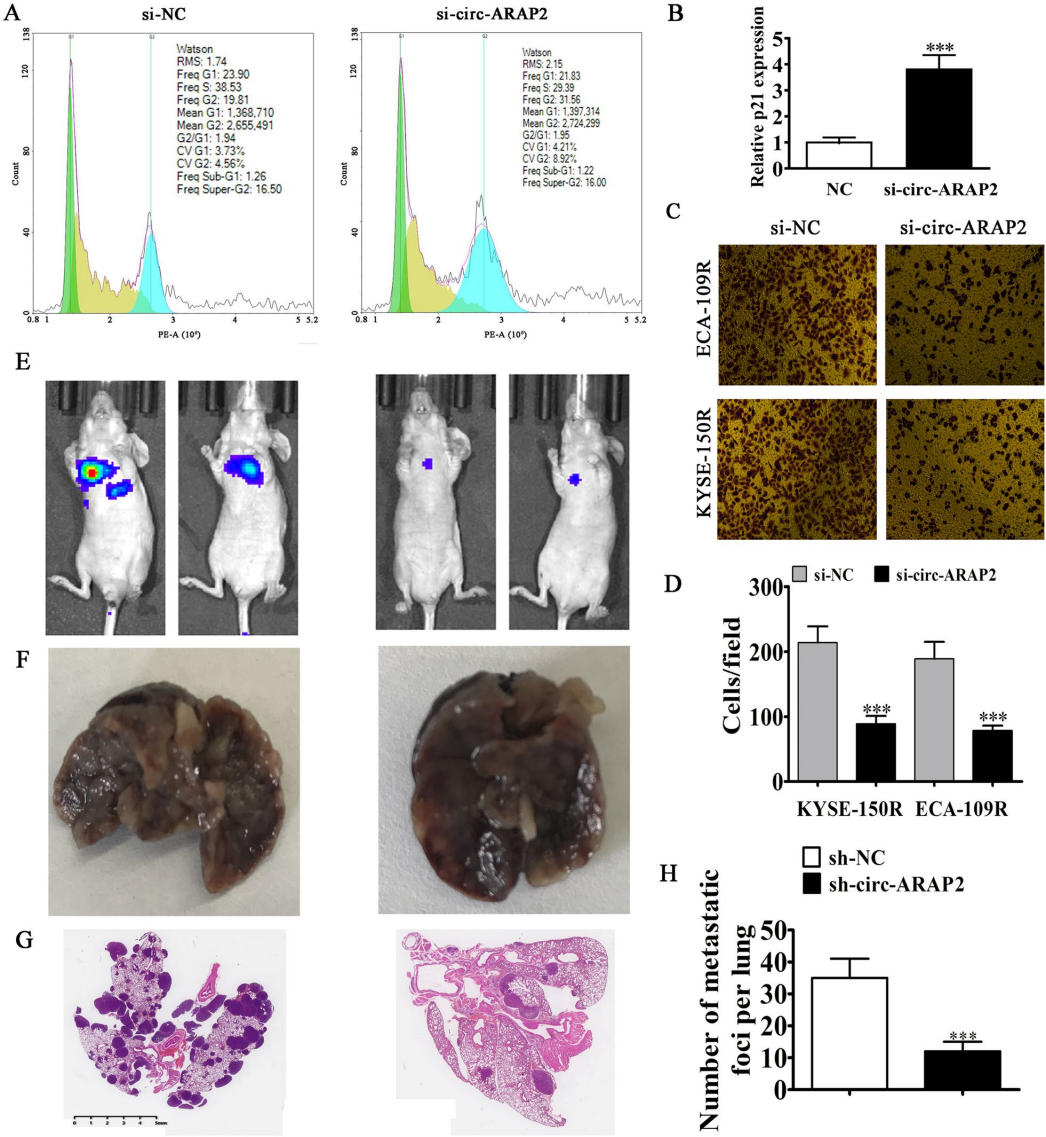
Knockdown of circ-ARAP2 suppressed ESCC metastasis. **A** Representative results showing the percentage of cells in G1, S, or G2 phase in ECA-109R cells by flow cytometry. **B** RT-qPCR were used to detected the expression of p21. Data are presented as the mean ± SD. ^***^p < 0.001 vs. NC. **C**, **D** Cellular migration was assessed in both KYSE-150R and ECA-109R cells using transwell assays. Data are presented as the mean ± SD. ^***^p < 0.001 vs. si-NC. **E** Living imaging detection show the ECA-109R cells pulmonary metastasis. **F**–**H** The numbers of metastatic foci in lung tissues were caculation according to the HE staining. The data are expressed as the mean ± SD. ^***^p < 0.001 vs sh-NC

Cell cycle analyses using flow cytometry were conducted on ECA-109R cells and showed that circ-ARAP2 knockdown led to partial cell cycle arrest in G2 phase by approximately 12% (Fig. 4A). RT-qPCR detection show the expression of cell cycle relative protein p21 and found that circ-ARAP2 silence promoted p21 expression (Fig. 4B). Our team investigated the circ-ARAP2 effects on ESCC invasiveness. The in vitro experiment utilizing transwell assays found that knockdown of circ-ARAP2 suppressed cell migration of both KYSE-150R and ECA-109R cells (Fig. 4C, D). Intravenous tail injection of KYSE-150R cells with or not circ-ARAP2 knockdown showed by live imaging analysis that 30 days after injection, KYSE-150R metastasis was decreased in circ-ARAP2-silenced mice (Fig. 4E). Lung metastatic nodule numbers were also diminished in the circ-ARAP2-silenced group (Fig. 4F–H). These results advised that circ-ARAP2 knockdown suppressed ESCC invasive ability in vitro and in vivo.

### The miR-761 and FOXM1 were circ-ARAP2 downstream targets

**Fig. 5:**
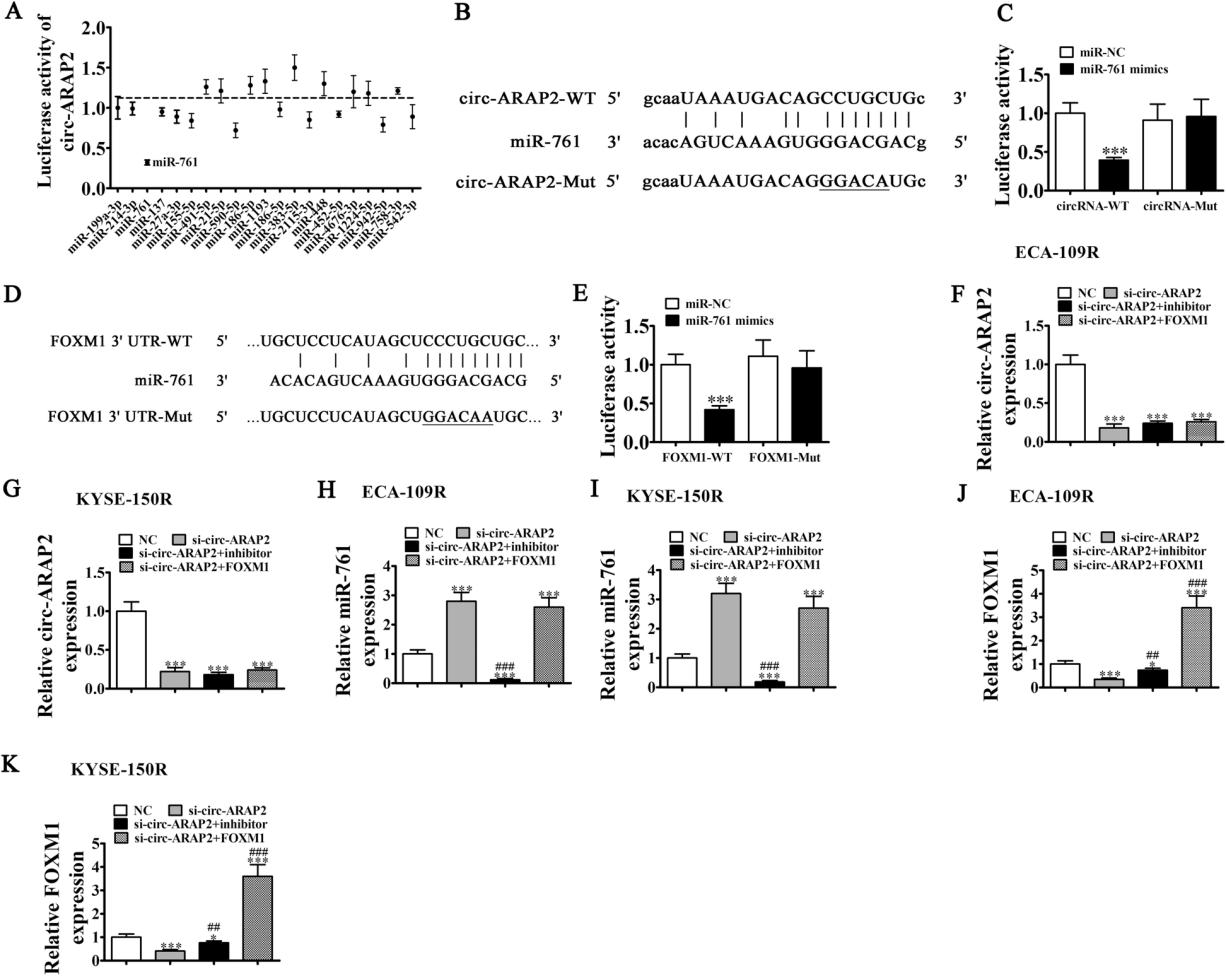
MiR-761 and FOXM1 were the downstream target of circ-ARAP2. **A** The luciferase activity of circ-ARAP2 in HEK293T cells transfected with different miRNA mimics, which are putative binding sites for the circ-ARAP2 sequence. Luciferase activity was normalized by Renilla luciferase activity. **B** Prediction of binding sites of miR-761 in circ-ARAP2. The MUT version of circ-ARAP2 is presented. **C** Relative luciferase activity determined 48 h after transfection of HEK293T cells with miR-761 mimic/NC or circ-ARAP2 WT/Mut. Data are presented as means ± SD. ***p < 0.001. **D** Prediction of binding sites of miR-761 within the 3'UTR of FOXM1. The MUT version of 3'-UTR-FOXM1 is shown. **E** Relative luciferase activity determined 48 h after transfection of HEK293T cells with miR-761 mimic/NC or 3'UTR-FOXM1 WT/Mut. Data are presented as means ± SD. ***p < 0.001. **F**–**K** RT-qPCR detection showing the expression of circ-ARAP2, miR-761 and FOXM1. Data are presented as mean ± SD; ^*^p < 0.05, ^***^p < 0.001 vs. NC; ^##^p < 0.01, ^###^p < 0.001 vs. si-circ-ARAP2

Bioinformatics analysis revealed that circ-ARAP2 interacted with a series of miRNAs including miR-199a-3p, miR-214-3p, miR-761, miR-27a-3p, miR-491-5p, miR-186-5p, miR-383-5p, and others. We constructed a luciferase reporter vector containing the circ-ARAP2 sequence and co-transfected it with different miRNA mimics into HEK293 cells. These result showed that only transfection of miR-761 significantly decreased fluorescein intensity, suggesting that miR-761 was circ-ARAP2 downstream target (Fig. 5A). Luciferase reporter assays further validated that miR-761 inhibited circ-ARAP2 in WT cells, yet not in Mut cell lines (Fig. 5B, C), illustrating that miR-761 was the circ-ARAP2 target.

Bioinformatics analyses informed that FOXM1 was the miR-761 downstream target. High-throughput sequencing discovered that FOXM1 expression incremented in tumor tissues comparing to matched non-tumorous tissues (Additional file 2). To verify correlations between miR-761 and FOXM1, Mut or WT 3'-UTR-FOXM1 sequences incorporating the miR-761 binding sequence were constructed in luciferase reporter vectors (Fig. 5E). These constructs were then co-transfected into HEK293 cells with or not the miR-761 mimic. Luciferase reporter results illustrated that miR-761 inhibited luciferase activity in WT cells, yet not in Mut cell lines (Fig. 5F), illustrating that FOXM1 was the miR-761 target.

RT-qPCR data demonstrated that circ-ARAP2 expression was diminished after transfection with the circ-ARAP2 silencing vector, while treatment with miR-761 inhibitor or overexpressing FOXM1 can not restore circ-ARAP2 expression in both KYSE-150R and ECA-109R cells (Fig. 5E, F), which indicated that both miR-761 and FOXM1 were downstream targets of circ-ARAP2. RT-qPCR detection also found that circ-ARAP2 silencing increased miR-761 expression, while FOXM1 overexpression did not influence si-circ-ARAP2-induced miR-761 expression (Fig 5H, I), again suggesting that miR-761 was circ-ARAP2 downstream target. We also observed that circ-ARAP2 silencing decreased FOXM1 expression, but down-regulation of miR-761 reversed the inhibitory effect of si-circ-ARAP2 on FOXM1 expression. After transfection with the FOXM1 overexpression vector, FOXM1 expression increased significantly (Fig. 5J, K), which suggested that circ-ARAP2 promoted FOXM1 expression via sponging miR-761.

### FOXM1 overexpression or miR-761 inhibition reversed invasion and proliferation by ESCC cells induced by silencing circ-ARAP2

**Fig. 6:**
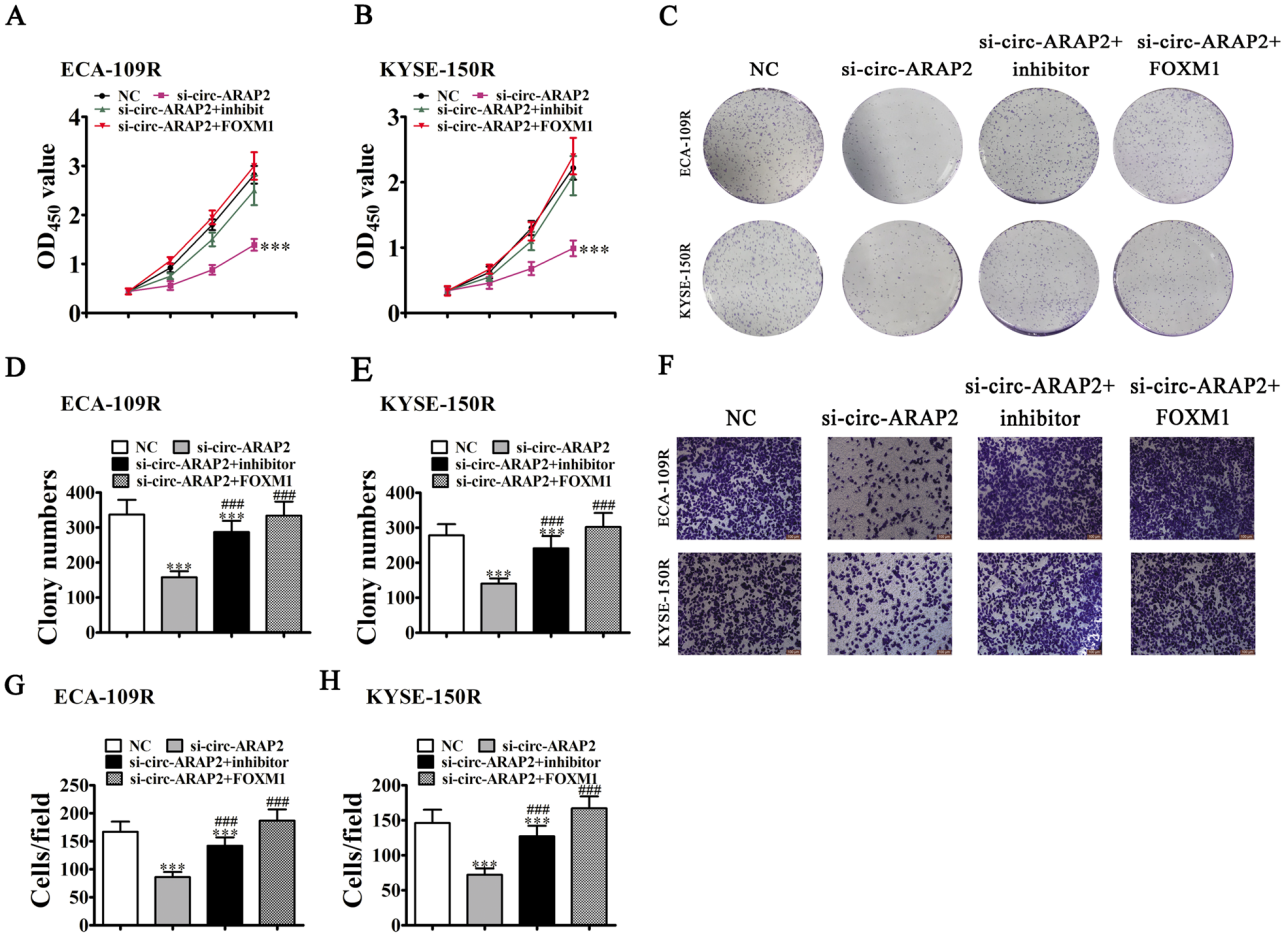
Overexpression of FOXM1 or inhibit miR-761 reversed ESCC cells proliferation and invasion after silence circ-ARAP2. **A**, **B** CCK8 detection show the proliferation ability of KYSE-150R and ECA-109R cells. Data are presented as mean ± SD; ^***^p < 0.001 vs. NC. **C**–**E** Cloning formation assay showing the cellular proliferation in both KYSE-150R and ECA-109R cells. Data are presented as mean ± SD; ^***^p < 0.001 vs. NC; ^###^p < 0.001 vs. si-circ-ARAP2. **F**–**H** Transwell detection show the invasion and migration of KYSE-150R and ECA-109R cells. Data are presented as mean ± SD; ^***^p < 0.001 vs. NC; ^###^p < 0.001 vs. si-circ-ARAP2

In vitro experiments using CCK8 (Fig. 6A, B) and clonal formation assays (Fig. 6C–E) showed that miR-761 inhibitor treatment or overexpression of FOXM1 restored cell activity/proliferation after circ-ARAP2 knockdown in both KYSE-150R and ECA-109R cells. Transwell migration analysis also found that miR-761 down-regulation or FOXM overexpression restored cell migration after circ-ARAP2 knockdown in both KYSE-150R and ECA-109R cells. These results indicated that FOXM1 overexpression or inhibition of miR-761 reversed ESCC cell proliferation and invasion after silencing circ-ARAP2.

### The circ-ARAP2 influences the endothelial–mesenchymal transition (EMT) and cancer stem cell differentiation through regulating miR-761/FOXM1

**Fig. 7:**
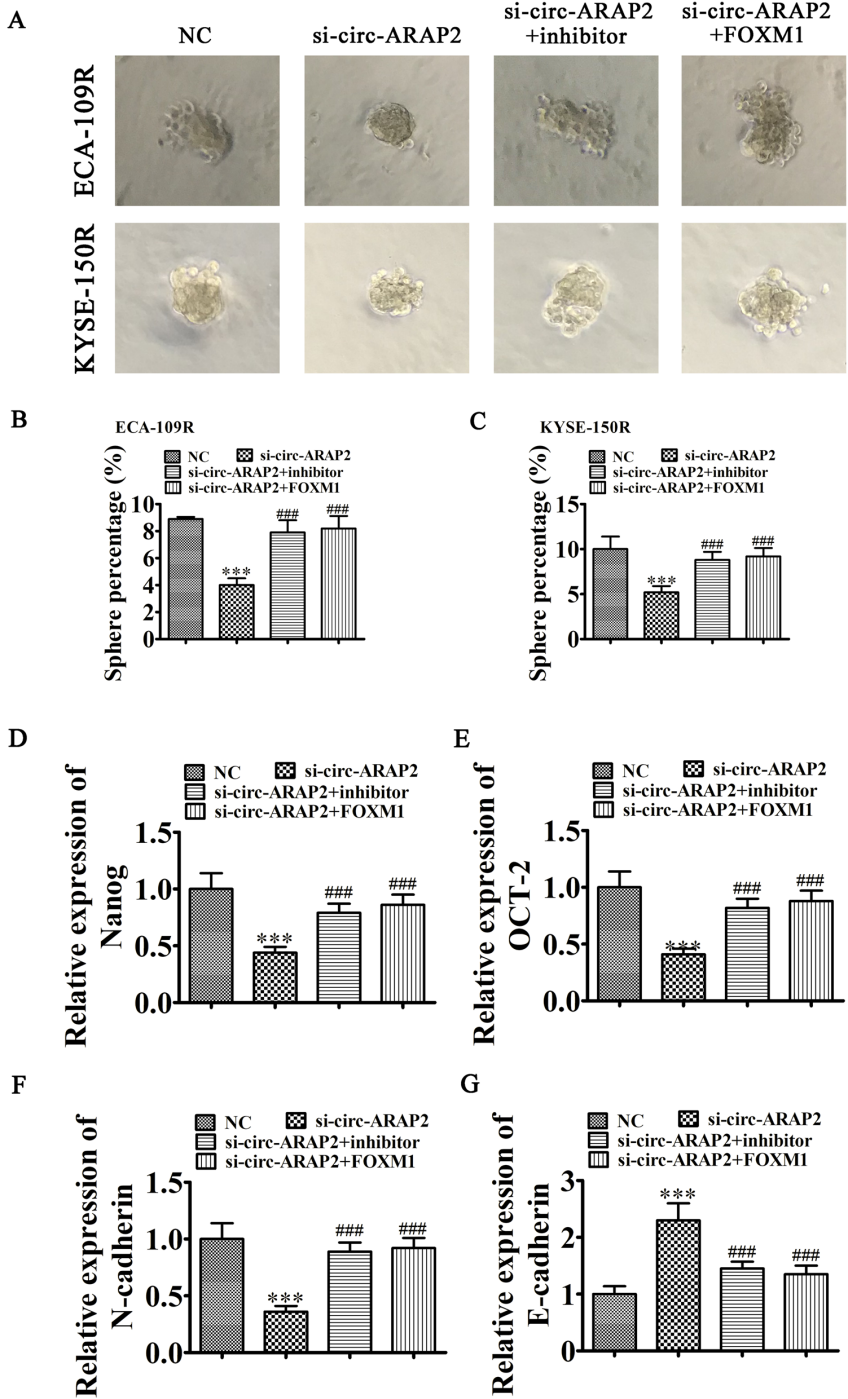
circ-ARAP2 can influence EMT and cancer stem cells different by regulation miR-761/FOXM1. **A**–**C** Images of tumor sphere formation assays in KYSE-150R and ECA-109R cell. Data are presented as mean ± SD; ^***^p < 0.001 vs. NC; ^###^p < 0.001 vs. si-circ-ARAP2. **D**, **E** RT-qPCR detection show the expression of stemness markers OCT4 and Nanog in KYSE-150R cells. Data are presented as mean ± SD; ^***^P < 0.001 vs. NC; ^###^p < 0.001 vs. si-circ-ARAP2. **F**, **G** RT-qPCR detection shows the relative expression of EMT relative E-cadherin and N-cadherin expression. Data are presented as mean ± SD; ^***^p < 0.001 vs. NC; ^###^p < 0.001 vs. si-circ-ARAP2

Tumor sphere formation assays using KYSE-150R and ECA-109R cells showed that circ-ARAP2 silencing decreased cell division. In contrast, overexpression of FOXM1 or inhibition of miR-761 restored tumor sphere formation ability (Fig. 7A–C). FACS detection with CD44 staining also found that overexpression of FOXM1 or inhibition of miR-761 restored CSCs formation ability of KYSE-150R after silience circ-ARAP2 (Additional file 3). RT-qPCR demonstrated that circ-ARAP2 silencing decreased stemness marker OCT4 expression and Nanog in KYSE-150R cells, but overexpression of FOXM1 or inhibition of miR-761 restored their expression (Fig. 7D, E). RT-qPCR detection also found that down-regulation of miR-761 or overexpression of FOXM1 rescued EMT-related N-cadherin and E-cadherin expression in KYSE-150R cells after circ-ARAP2 silencing (Fig. 7F, G). These results suggested that circ-ARAP2 influenced EMT and cancer stem cell gene expression via regulation of miR-761/FOXM1.

## Discussion

Though circRNA biogenesis and expression are studied intensively recently, the circRNA functions in cancer remains unknown [18, 19]. Current research discovered that several circRNA expressions are enhanced in ESCC, and circ-ARAP2 in particular is significantly incremented in ESCC tissues compared with adjacent non-tumorous tissues. Our data better indicated that circ-ARAP2 down-regulation significantly decreased ESCC invasion and proliferation in vivo and in vitro. Additional experiments demonstrated that miR-761 was circ-ARAP2 downstream target. Luciferase reporter analysis verified correlation between miR-761 and circ-ARAP2, while RT-qPCR assays demonstrated that circ-ARAP2 silencing promoted miR-761 expression. Previous research studies have reported that miR-761 expression has an inhibitory effect on cancer invasion and proliferation in colorectal cancer [20, 21], glioma [22], gastric cancer [23], papillary thyroid cancer [24] and triple-negative breast cancer [25]. While the miR-761 role in ESCC remains unknown. Our team discovered that inhibiting miR-761 reversed ESCC cell proliferation and invasive ability after circ-ARAP2 was silenced.

Additional experiments revealed that FOXM1 expression in ESCC tissues was increased, and bioinformatics and luciferase reporter analysis validated that miR-761 interacted with the FOXM1 3'-UTR. Down-regulation of circ-ARAP2 decreased FOXM1 expression, but inhibiting miR-761 reversed FOXM1 expression after silencing circ-ARAP2. Previous research demonstrated that elevated FOXM1 levels enhance cancer progression and are associated with various aggressive and chemotherapy-resistant human cancers [26–28]. Emerging evidence supports that cancer stem cell (CSCs) and EMT function importantly in cancer progression and shows they are involved in recurrence, metastasis and drug resistance of various tumors [29–31]. We also found that down-regulation of miR-761 or overexpression of FOXM1 restored CSC gene expression and EMT-related protein expressions N-cadherin and E-cadherin after circ-ARAP2 was silenced.

**Fig. 8:**
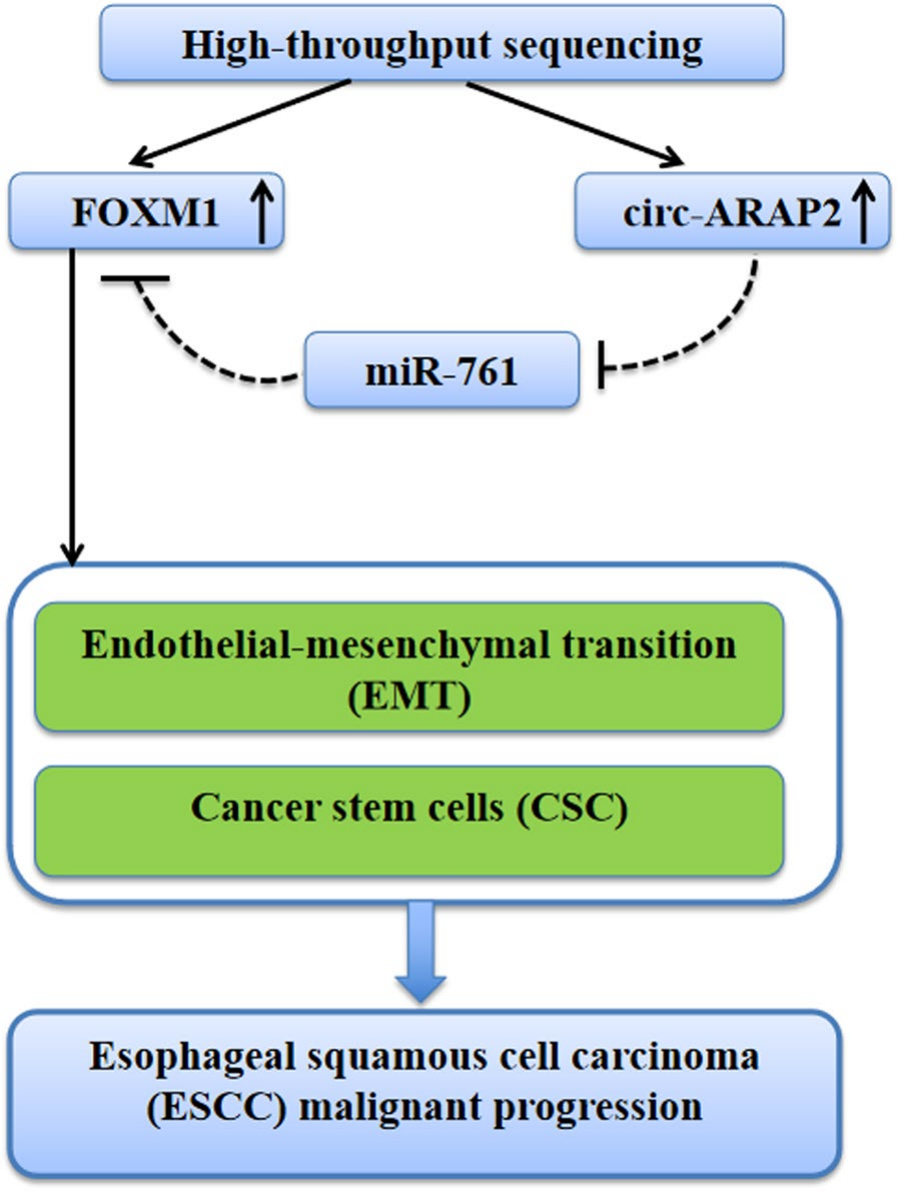
circ-ARAP2 promotes cancer progression via endothelial–mesenchymal transition (EMT) and cancer stem cells differently by regulating miR-761/FOXM1

In conclusion, the increase in circ-ARAP2 expression played a role in ESCC development. Our data demonstrated that circ-ARAP2 directly targeted miR-761 via FOXM1 up-regulation. The circ-ARAP2 down-regulation suppressed ESCC progression via incrementing miR-761 and decrementing FOXM1 expression (Fig. 8). The data suggested novel ESCC treatment targets which deserve more study.

## Supplementary Information

**Additional file 1.** High-throughput sequencing for circRNA expression detection.

**Additional file 2.** High-throughput sequencing for mRNA expression detection.

**Additional file 3.** The apoptosis detection using flow cytometry.

## Data Availability

All data in the article are available.
