# Supplementary figures and images for: The abnormal expression of circ-ARAP2 promotes ESCC progression through regulating miR-761/FOXM1 axis-mediated stemness and the endothelial–mesenchymal transition
Source: J Transl Med. 2022 Jul 16;20:318. doi: 10.1186/s12967-022-03507-3 (PMC9287963; doi:10.1186/s12967-022-03507-3)

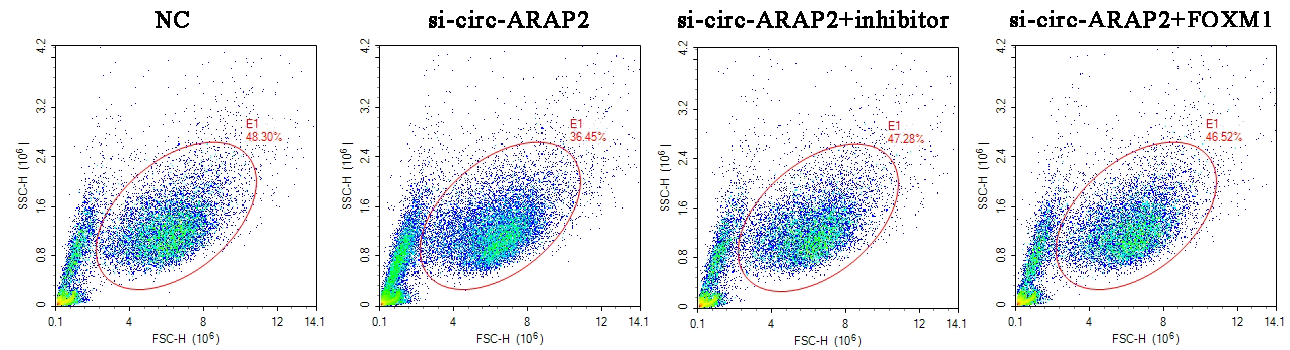

Supplement: Supplementary file 3 — Additional file 3. The apoptosis detection using flow cytometry. [file 12967_2022_3507_MOESM3_ESM.tif]
